# High mobility group box 1 (HMGB1): a pivotal regulator of hematopoietic malignancies

**DOI:** 10.1186/s13045-020-00920-3

**Published:** 2020-07-13

**Authors:** Shunling Yuan, Zhaoping Liu, Zhenru Xu, Jing Liu, Ji Zhang

**Affiliations:** 1 Department of Clinical Laboratory, The First Affiliated Hospital, University of South China, Hengyang, 421001 Hunan China; 2 Hunan Province Key Laboratory of Basic and Applied Hematology, Molecular Biology Research Center & Center for Medical Genetics, School of Life Sciences, Central South University, Changsha, 410078 Hunan China

**Keywords:** High mobility group box 1 (HMGB1), Hematopoietic stem cells (HSCs), Bone marrow (BM) microenvironment, Inflammation, Chemoresistance

## Abstract

High mobility group box 1 (HMGB1) is a nonhistone chromatin-associated protein that has been widely reported to play a pivotal role in the pathogenesis of hematopoietic malignancies. As a representative damage-associated molecular pattern (DAMP), HMGB1 normally exists inside cells but can be secreted into the extracellular environment through passive or active release. Extracellular HMGB1 binds with several different receptors and interactors to mediate the proliferation, differentiation, mobilization, and senescence of hematopoietic stem cells (HSCs). HMGB1 is also involved in the formation of the inflammatory bone marrow (BM) microenvironment by activating proinflammatory signaling pathways. Moreover, HMGB1-dependent autophagy induces chemotherapy resistance in leukemia and multiple myeloma. In this review, we systematically summarize the emerging roles of HMGB1 in carcinogenesis, progression, prognosis, and potential clinical applications in different hematopoietic malignancies. In summary, targeting the regulation of HMGB1 activity in HSCs and the BM microenvironment is highly beneficial in the diagnosis and treatment of various hematopoietic malignancies.

## Introduction

High mobility group (HMG) is a non-histone chromosome-binding protein in eukaryotic cells that is named after its low molecular weight and high gel mobility [1]. According to the HMG molecular weight, structural similarity and DNA binding characteristics, HMG proteins are divided into three gene families: HMGA, HMGB, and HMGN. HMGB1, also known as amphoterin or HMG1, is the most abundant nonhistone nucleoprotein in the HMGB gene family. HMGB1 is also expressed to some extent in the cytoplasm, as it shuttles back and forth from the nucleus [2]. HMGB1 has dual functions as a nonhistone nucleoprotein and an extracellular inflammatory cytokine. Intracellular HMGB1 is extensively bound to DNA and involved in transcriptional regulation, DNA replication and repair, telomere maintenance, and nucleosome assembly. Extracellular HMGB1 is passively released by necrotic tissue or stressed cells or actively secreted. As a chemokine or cytokine, it binds to pattern recognition receptors (PRRs) to play the role of a damage-associated molecular pattern (DAMP) [3].

## Overview of HMGB1

### The biological structure of HMGB1

**Fig. 1:**
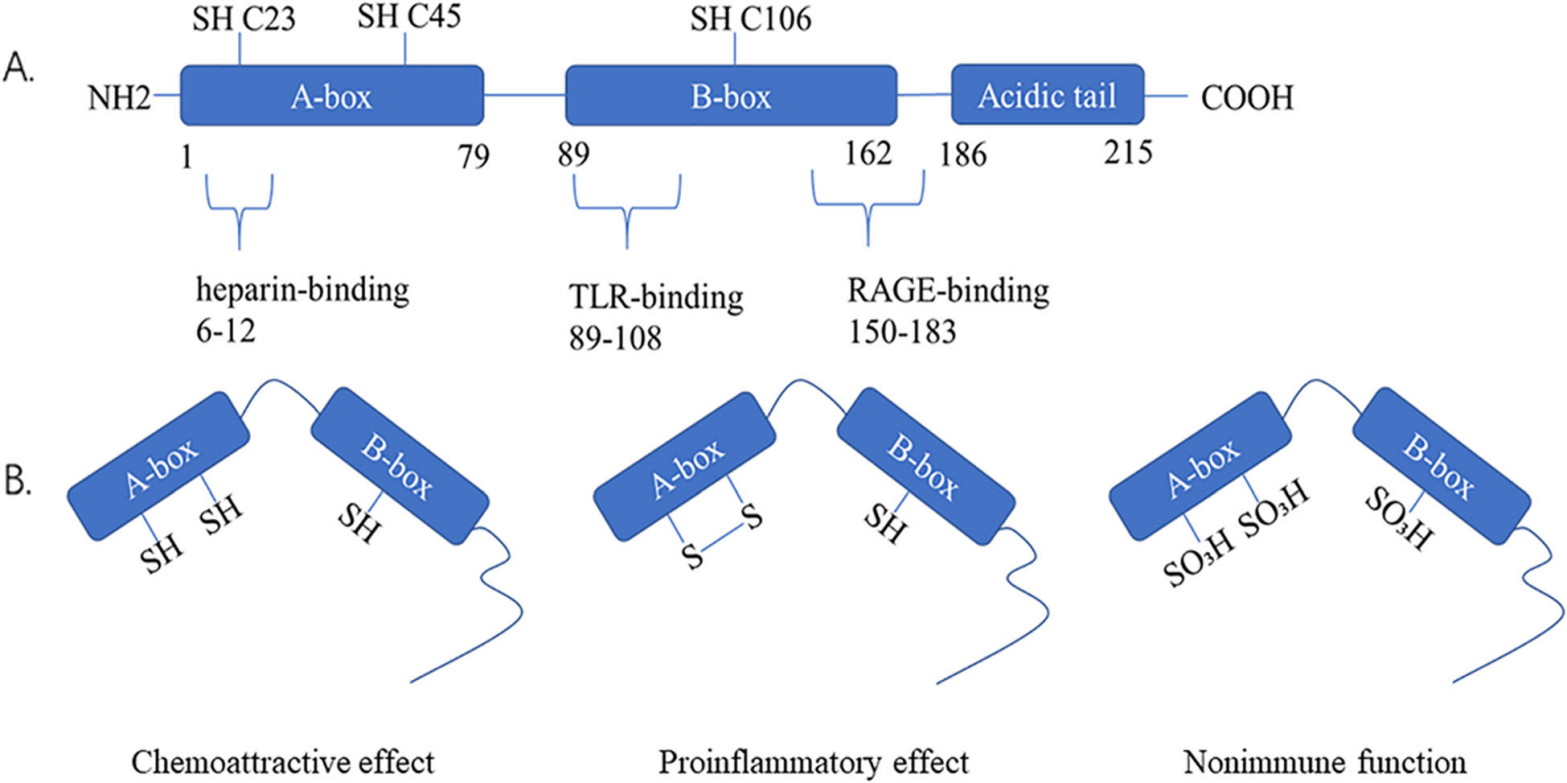
The structure and redox modifications of the HMGB1 protein. **a** The human HMGB1 protein is composed of 215 amino acid residues and is divided into three functional domains: the A-box, B-box, and acidic C-terminal tail. Three redox-sensitive cysteine residues at the 23rd, 45th, and 106th positions regulate HMGB1 functions in response to oxidative stress. The three ligand binding sites of the HMGB1 protein and activate signaling mechanisms: heparin binding site (6-12 aa), TLR4 binding site (89-108 aa), and RAGE binding site (150-183 aa). **b** There are three structural arrangements of the HMGB1 protein, reduced state, oxidized state and disulfide bond formed structure, which result in HMGB1 acting as a chemokine, an inflammatory factor and a nonimmune factor, respectively

The HMGB1 gene is located on chromosome 13q12 and includes five exons and four introns. The TATA box promoter of the HMGB1 gene contains binding sites for several transcription factors, such as activator protein 1 (AP1), and a silencing element [4]. Human HMGB1 protein is a highly conserved nuclear protein consisting of 215 amino acids with a molecular weight of approximately 30 kD. Structurally, HMGB1 is divided into three functional regions (Fig. 1): A-box (9-79 aa), B-box (89-162 aa), and acidic C-terminus (186-215 aa). The A-box and B-box are composed of 80–90 amino acid residues, with similar amino acid repeats and nonspecific DNA binding sites; the B-box is a functional structural region that causes an inflammatory response [5]. However, the A-box has a certain antagonistic effect on the B-box [6]; the acidic C-terminus containing aspartic acid and glutamic acid is mainly involved in regulating the binding affinity between HMGB1 and DNA, and mediates gene transcription and chromosome derotation [7]. The N-terminus of HMGB1 (6–12 aa) contributes to heparin-binding activity. After binding to HMGB1, heparin impacts the spatial conformation of HMGB1, reduces the affinity of HMGB1 for its receptor, and inhibits its proinflammatory activity [8, 9]. The B-box domain has two crucial binding sites for Toll-like receptor 4 (TLR4) and receptor for advanced glycation end products (RAGE), which regulate the release of proinflammatory cytokines. The RAGE binding site of HMGB1 is located between amino acid residues 150 and 183, and the 20 amino acids of the TLR4 binding site (89-–108 aa) are the minimal sequence necessary to induce cytokine activity [10, 11]. Although HMGB1 is an evolutionarily conserved multifunctional protein, the biological function of HMGB1 depends on its modifications, cellular location, redox state, and binding partners.

### Posttranslational modification of HMGB1

The HMGB1 protein shuttles between the nucleus and cytoplasm because it contains two nuclear localization sequences (NLSs) and two putative nuclear export signals (NESs). HMGB1 interacts with the nuclear receptor chromosome-region maintenance-1 (CRM-1), which is a nuclear transport receptor involved in the export of leucine-rich NES proteins and is then released from the nucleus into the cytoplasm [12]. The conserved lysine residues in the NLSs are sensitive to acetylation and can activate nuclear exclusion and HMGB1 translocation [13–15]. In addition to acetylation, HMGB1 is regulated by extensive posttranslational modifications (PTMs) including methylation, phosphorylation, ADP-ribosylation, glycosylation, and ubiquitination. These PTMs redirect it toward secretion and modulate its interactions with DNA and other proteins [16]. Then, the oxidation of extracellular HMGB1 determines its bioactivity in mediating inflammation and innate immune responses.

#### Acetylation

HMGB1 is usually located in the cell nucleus. Since HMGB1 lacks a secretory signal peptide and does not traverse the ER-Golgi system, the secretion of this nuclear protein seems to require a tightly controlled relocation program [17]. Numerous studies have proven that acetylation regulates the cytoplasmic accumulation of HMGB1. In the inflammatory response, HMGB1 is extensively acetylated in monocytes and macrophages upon activation with lipopolysaccharide; moreover, enhanced hyperacetylation of HMGB1 in resting macrophages causes HMGB1 translocation to the cytoplasm. Cytosolic HMGB1 is concentrated by default into secretory lysosomes and secreted when monocytic cells receive the appropriate second signal. P300/CBP-associated factor (PCAF), CREB-binding protein (CBP), and histone acetyltransferase p300 (p300) play important roles in HMGB1 acetylation [13]. Mass spectrometric analysis revealed that type 1 interferon (IFN)-stimulated activation of JAK/signal transducer and activator of transcription 1 (STAT1) could induce HMGB1 acetylation and translocation from the nucleus to the cytoplasm [18].

#### Methylation

Besides acetylation, it has been demonstrated that the monomethylation of lysine-42 in HMGB1 isolated from neutrophils regulates its relocalization from the nucleus to the cytoplasm. Methylated HMGB1 is mostly located in the cytoplasm of neutrophils, while unmethylated HMGB1 is present in the nucleus. Because methylation leads to conformational changes in the HMGB1 protein, the possible mechanism by which methylation controls distribution is that methylation of Lys-42 alters the conformation of the A-box, thereby impairing its ability to bind to DNA. Then, methylated HMGB1 passively diffuses from the nucleus into the cytoplasm [19].

#### Phosphorylation

Phosphorylation is also important in blocking HMGB1 re-entry to the nucleus and accumulating in the cytoplasm. Earlier reports found that HMGB1 isolated from lamb thymus could be phosphorylated by calcium/phospholipid-dependent protein kinase but not by cAMP-dependent protein kinase [20]. Recently, a study demonstrated that HMGB1 was phosphorylated in RAW264.7 cells and human monocytes after treatment with tumor necrosis factor alpha (TNF-α) or okadaic acid (OA, a phosphatase inhibitor), resulting in the transport of HMGB1 to the cytoplasm and eventual secretion. The six possible phosphorylation sites are Ser-34, Ser-38, Ser-41, Ser-45, Ser-52, and Ser-180, which are mainly around NLS1 and NLS2 [14]. Moreover, phosphorylation promotes HMGB1 relocation to the cytoplasm and subsequent secretion through protein kinase C-regulated calcium-dependent mechanisms [21].

#### ADP-ribosylation

ADP-ribosylation reactions add one or more ADP-ribose moieties to a protein by ADP-ribosyl transferases, and are classified into four groups: mono-ADP-ribosylation, poly-ADP-ribosylation, ADP-ribose cyclization, and the formation of *O*-acetyl-ADP-ribose. Hyper ADP-ribosylation of HMGB1 downregulates gene transcription since ADP-ribosylation is generally inversely related to transcription. Recently, the poly-ADP-ribosylation of HMGB1 was found to facilitate its acetylation and promoted HMGB1 translocation-associated chemotherapy-induced autophagy in leukemia cells [22]. The activation of SIRT6 and PARP1 is required for chemotherapy-induced ADP-ribosylation of HMGB1 and mediates HMGB1 translocation [23]. Hyperpoly-ADP-ribosylation of HMGB1 enhances the inhibition of efferocytosis, but a lack of intracellular HMGB1 leads to excessive activation and damage of PARP1 [24, 25]. Hence, HMGB1 and PARP1 can regulate cell death by ADP-ribosylation.

#### Glycosylation

HMGB1 *N*-glycosylation plays a prerequisite role in nucleocytoplasmic translocation and extracellular secretion. HMGB1 was reported to be *N*-glycosylated at Asn-37 and alternatively at Asn-134/135 residues, which determines HMGB1 nucleocytoplasmic transport, extracellular secretion, and protein stability. Moreover, two *N*-glycosylations at Asn-37 and Asn-134 were further identified as the consensus motifs of Asn-Xxx-Ser/Thr, whereas recombinant HMGB1 protein was glyecosylated at the noncelassical consensus residue Asn-135 in both HEK293T and insect cells [26].

#### Ubiquitination

Protein ubiquitination participates in many basic cellular processes, such as proteolysis, DNA repair, and DNA transcription, in response to diverse stimuli [27]. Ubiquitin (Ub) is an evolutionarily conserved protein that posttranslationally marks proteins for degradation [28]. It has been reported that the enhanced level of HMGB1 ubiquitination may be the causative factor in multiple myeloma (MM). Moreover, MALAT-1 knockdown promotes the degradation of HMGB1 at the posttranslational level by increasing the ubiquitination of HMGB1 in MM cells [29]. It was also found that lycorine downregulates HMGB1 by promoting HMGB1 ubiquitination to inhibit autophagy in MM cells [30]. This finding suggests that ubiquitin proteasome system (UPS) inhibitors could have great therapeutic potential for MM treatment in the clinic.

#### Oxidation

HMGB1 contains three cysteine residues at positions 23rd, 45th, and 106th that are susceptible to redox-dependent modifications. When released into the extracellular space, HMGB1 is initially in a fully reduced state (fr-HMGB1) but becomes disulfide-HMGB1 (ds-HMGB1) due to the oxidative environment. When exposed to a large amount of reactive oxygen species (ROS) from activated leukocytes, HMGB1 can be sulfonated (ox-HMGB1). These three different extracellular HMGB1 redox states play distinct roles in inflammation. fr-HMGB1 binds to CXC motif ligand (CXCL) 12 and stimulates chemoattraction via the CXC motif chemokine receptor type 4 (CXCR4) [31]. Under normal circumstances, the majority of intracellular HMGB1 is fully reduced, which maintains structural integrity and protects against terminal oxidation by ROS [32]. Reduced cysteine residues also make HMGB1 a chemoattractant that can recruit leukocytes and promote tissue regeneration [33, 34]. ds-HMGB1 has a disulfide bond between cysteine 23 and cysteine 45, which elicits inflammatory responses and cytokine-inducing activity through TLR4/myeloid differentiation factor 2 (MD-2) [35]. In ox-HMGB1, the cysteines are fully oxidized or C-106 is oxidized, preventing HMGB1 from having cytokine or chemotactic activity. Furthermore, ox-HMGB1 participates in the resolution of inflammation in highly acidic conditions [36]. The redox status of HMGB1 in terms of location and release directly influences its extracellular activity, such as immunity and inflammation [32].

### The release mechanism of HMGB1

**Fig. 2:**
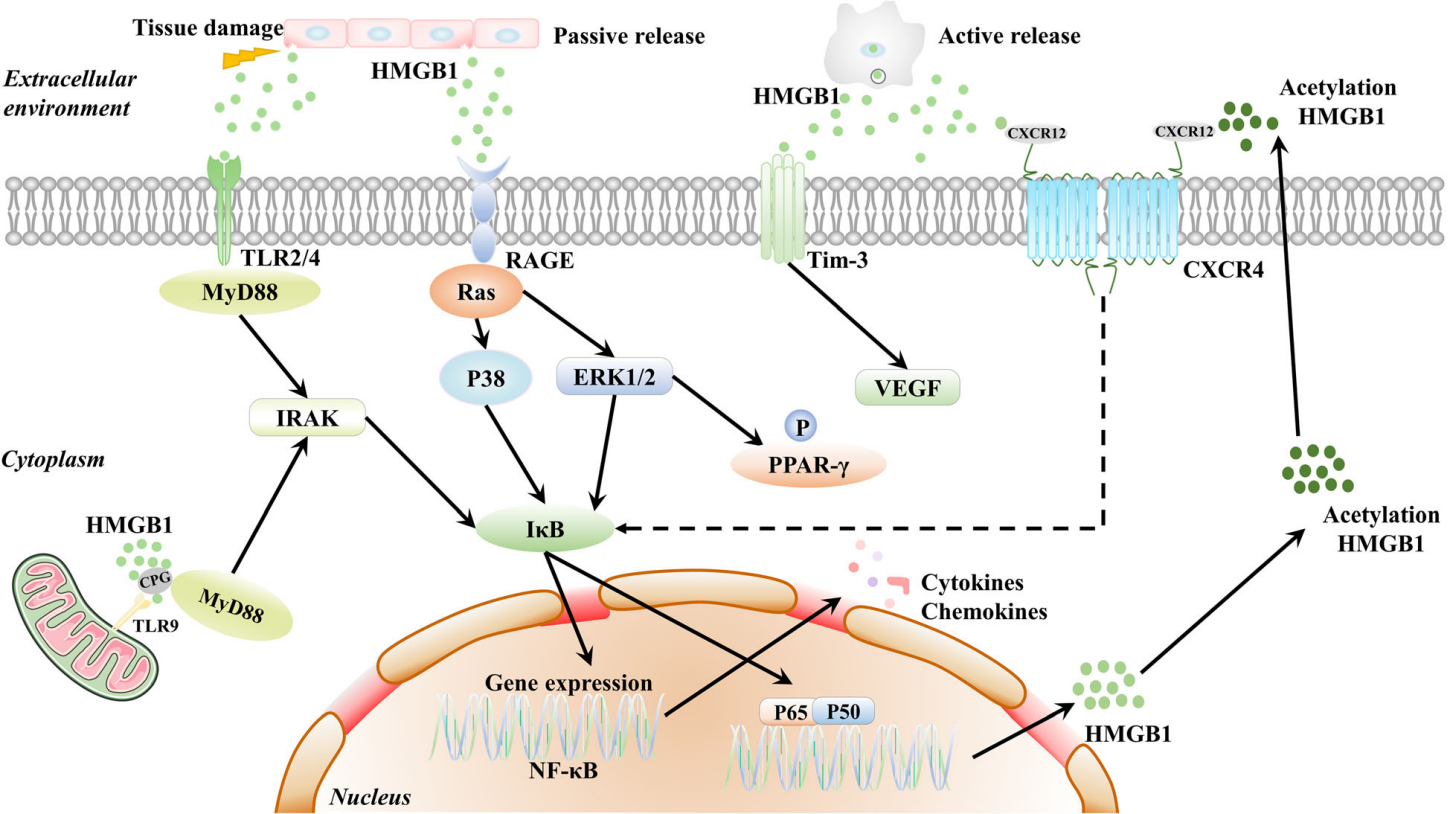
The release of HMGB1 protein and HMGB1 signaling pathways. The release mechanism of HMGB1 into the extracellular environment includes passive release and active release. In response to infections and injuries, HMGB1 can translocate outside the cell by passive release from damaged or necrotic cells or active secretion from activated immune cells. The interaction of HMGB1 with RAGE, TLR2, TLR4, and TLR9 transduces cellular signals through a common pathway that induces the NF-κB pathway. Then, activated NF-κB translocates to the nucleus and interacts with DNA as a p65/p50 heterodimer. HMGB1 also interacts with CXCL12/CXCR4 to activate the NF-κB pathway and induce chemotaxis and recruitment of inflammatory cells. The activated NF-κB pathway promotes nuclear HMGB1 acetylation and secretion. HMGB1 binding to RAGE could activate PPAR-γ, which could inhibit HMGB1-RAGE activation. The interaction of HMGB1 and TIM-3 induces the secretion of VEGF to promote tumor angiogenesis

There are two mechanisms for releasing HMGB1 into the extracellular environment: passive release and active release (Fig. 2). In response to infection and injury, HMGB1 can be actively secreted from activated immune cells or passively released from damaged or necrotic cells and transferred outside the cell [37, 38]. Active release of HMGB1 from macrophages or monocytes requires a proinflammatory stimulus that could cause an immune response. Active HMGB1 release promotes neutrophil recruitment and macrophage release of proinflammatory cytokines, such as TNF-α and interleukin-6 (IL-6) and dendritic cell (DC) activation [39]. HMGB1 can be passively secreted from the nuclei of necrotic cells and damaged cells and then triggers inflammatory responses by functioning as necrotic cell death markers [36].

### Extracellular HMGB1 receptors and signaling pathways

Once released from the cells, HMGB1 binds to cell-surface receptors, inducing a reaction as a prototypical DAMP. Classic HMGB1 receptors include RAGE, TLRs (TLR2, TLR4, and TLR9), CXCR4, and T cell immunoglobulin mucin-3 (TIM-3) [40, 41].

#### RAGE

In 1995, it was first discovered that RAGE bound with HMGB1 [42]. RAGE is a member of the immunoglobulin superfamily and is a transmembrane receptor that binds to advanced glycation end products. RAGE contains one extracellular immunoglobulin variable (IgV) domain for ligand addition, two constant “C”-type extracellular domains, a transmembrane spanning domain, and a 43-amino acid cytosolic tail for RAGE-mediated intracellular signaling [43]. Several studies claimed that RAGE is an essential receptor for HMGB1-induced cell autophagy, immune responses, adhesion, and migration, which is carried out through the mitogen-activated protein kinase (MAPK), nuclear factor (NF)-κB, and mammalian/mechanistic target of rapamycin (mTOR) signaling pathways [44, 45]. The proinflammatory effect of the HMGB1-RAGE axis is significantly associated with the NF-κB pathway, which involves extracellular signal-regulated kinase 1 and 2 (ERK1/2), and p38 MAPK. Then, activated NF-κB translocates to the nucleus and interacts with DNA as a p65/p50 heterodimer, which enhances proinflammatory cytokine expression [46–48]. Although the role of the HMGB1-RAGE axis in cancer is not completely clear, HMGB1 is critical for directly activating RAGE or activating peroxisome proliferator-activated receptor gamma (PPAR-γ) pathway, and inhibiting HMGB1-RAGE activation, which might be a beneficial cancer therapeutic strategy [49].

#### TLRs

TLRs are PRRs that consist of extracellular leucine-rich repeats (LRRs) and a cytoplasmic Toll/interleukin-1 receptor (TIR) domain. The ligand binds to LRRs and activates signal transduction pathways through TIR domains with conserved adaptor molecules. Most TLRs signal through MyD88, while TLR3 utilizes TRIF, and TLR4 is the only receptor that utilizes both MyD88 and TRIF. TLRs play a critical role in the promotion of macrophage activation, cytokine release, and tissue damage. The underlying mechanism involves the MyD88-dependent and MyD88-independent pathways and activation of downstream factors such as MAPK and IFN regulatory factors [50, 51]. HMGB1 can interact with TLRs and then induce a series of cytokines and chemokines by triggering relevant signal transduction pathways [52]. In addition, HMGB1 forms complexes with partner molecules and then acts via the partner’s receptor [53]. HMGB1 binds to CpG-DNA and promotes its interaction with the DNA-sensing TLR9 receptor [54]. Extracellular HMGB1 activates RAGE or TLR4 and forms a heterocomplex with CXCL12 that strongly activates CXCR4, promoting inflammatory and pain signals [31, 55].

#### CXCR4

CXCR4 was known as a coreceptor that supported T lymphocyte-tropic HIV infection of permissive cells in 1996 [56]. CXCR4 is a G-protein-coupled seven-transmembrane receptor (GPCR) that is widely expressed in CD34^+^ hematopoietic stem cells (HSCs), lymphocytes, monocytes and macrophages, endothelial and epithelial cells, and cancer cells [57]. CXCL12 (stromal cell-derived factor-1, SDF-1), the CXCR4 ligand, is expressed by hematopoietic cells in the bone marrow (BM), facilitating the adhesion and survival of malignant clones. The CXCL12/CXCR4 axis is involved in tumor progression, angiogenesis, metastasis, and survival by activating multiple signaling pathways, such as ERK1/2, ras, p38 MAPK, PLC/MAPK, and SAPK/JNK [58, 59]. CXCL12/CXCR4 antagonists have shown encouraging results in reducing the enhanced survival and proliferation of leukemia cells and sensitizing leukemia cells to chemotherapy [60, 61]. During inflammation or tissue damage, extracellular fr-HMGB1 exerts chemotactic activity and enhances leukocyte recruitment by forming a heterocomplex with CXCL12 and binding to CXCR4 [31, 62, 63]. It has been found that the IKKα/noncanonical NF-κB pathway is required for sustained CXCL12/SDF-1 production to induce migration toward HMGB1, indicating that the heterocomplex of HMGB1 and CXCL12/SDF-1 may induce cell migration through the NF-κB pathway [64].

#### TIM-3

TIM-3 is a member of the TIM gene family of immunoregulatory proteins. It is composed of an extracellular IgV domain, a mucin-like domain, a transmembrane domain, and an intracellular cytoplasmic tail, which is involved in the recognition of phosphatidylserine (PtdSer) on the surface of apoptotic cells [65]. TIM-3 is associated with the regulation of immune responses in autoimmunity and cancer and is expressed on regulatory T cells (Treg cells), myeloid cells, natural killer (NK) cells, and mast cells. DC-derived TIM-3 interacts with HMGB1 to suppress the transport of nucleic acids into endosomal vesicles and reduces the therapeutic efficacy of DNA vaccination and chemotherapy by attenuating the immunogenicity of nucleic acids released from dying tumor cells [66]. Anti-TIM-3 monoclonal antibodies can improve the effectiveness of chemotherapy in mice or mice depleted of all DCs [67]. Furthermore, blocking both TIM-3 and programmed cell death 1 (PD1) can improve antitumor T cell responses in patients with advanced cancers [68]. HMGB1 combined with Tim-3 induces the secretion of angiogenic vascular endothelial growth factor (VEGF) and promotes tumor angiogenesis [69]. The combined induction of antitumor immunity by TIM-3 and HMGB1 has become a potential target for tumor immunogenic chemotherapy and development.

## The role of HMGB1 in bone marrow

### HMGB1 and hematopoietic stem cells

**Fig. 3:**
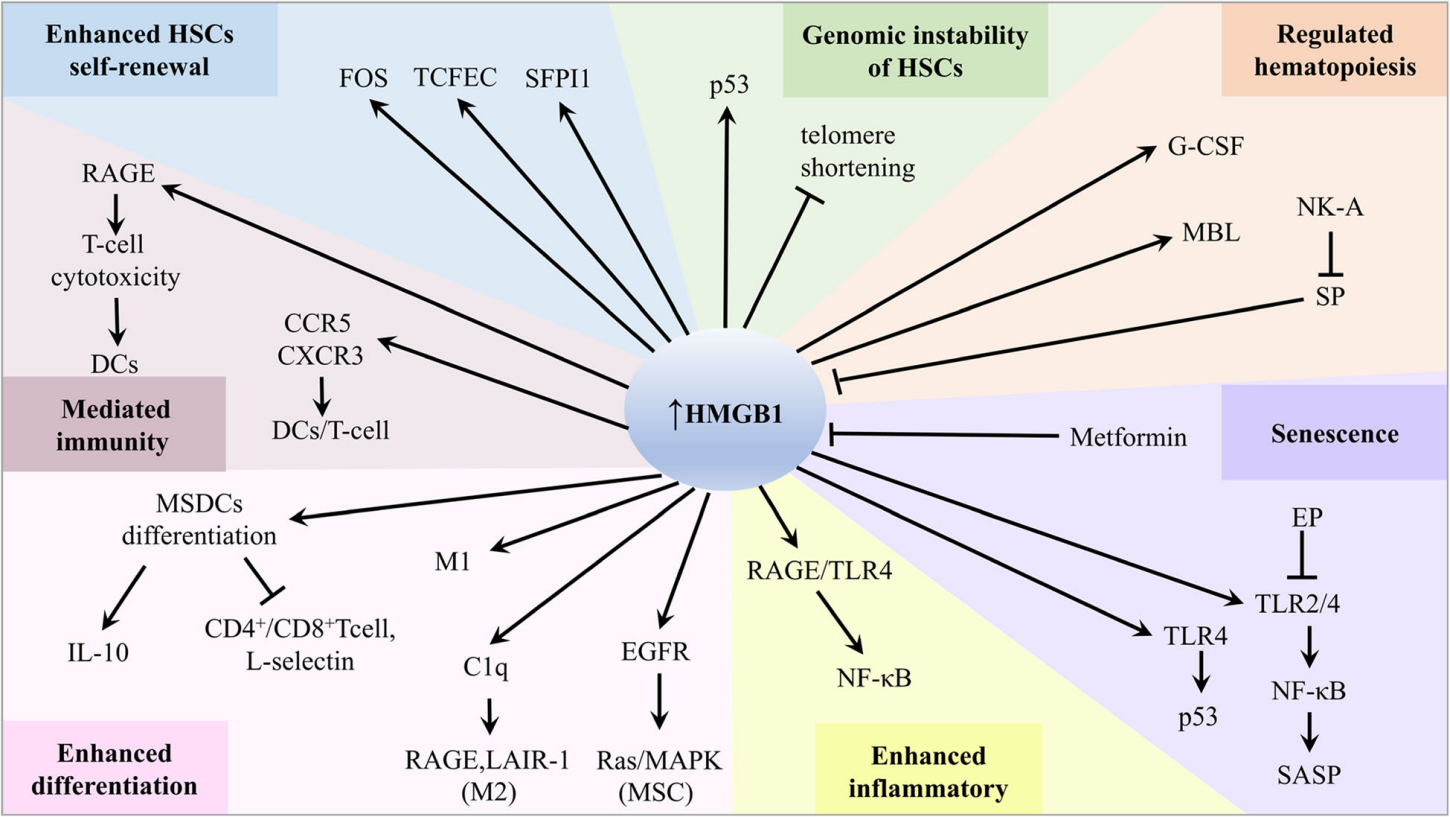
The roles of HMGB1 and associated molecules in BM. HMGB1 binds with a series of receptors or interactors and plays important roles in enhancing HSC self-renewal and differentiation, promoting senescence, regulating genomic instability, regulating hematopoiesis, mediating immunity, and affecting the inflammatory BM microenvironment

HMGB1 can regulate HSC multipotency and self-renewal at the transcriptional level (Fig. 3). In conjunction with FOS, TCFEC, and SFPI1, HMGB1 confers a clear repopulation advantage to HSCs via a non-cell-autonomous phenomenon [70]. A recent study demonstrated that HMGB1^−/−^ mouse embryonic fibroblasts (MEFs) showed slight telomere shortening but significantly decreased telomerase activity and DNA damage [71]. This indicates that HMGB1 may modulate chromosomal stability of HSCs by altering the functional chromatin structure of telomeres. HMGB1 can also bind to p53 DNA and stimulate DNA linearization, which increases p53 activity [72]. Moreover, the HMGB1 A-box has strong p53 binding activity based on crosslinking chemical and biophysical measurements [73]. HMGB1 regulates not only the transcriptional activity of p53 but also the subcellular localization and phosphorylation of p53.

HMGB1 plays an important role in the mobilization of HSPCs, thus regulating BM microenvironment formation. Altmann S et al. found that HMGB1 was broadly expressed in canine hematopoietic cells and directly induced the proliferation of peripheral blood mononuclear cells (PBMCs) [74]. Furthermore, mobilization of HSPCs is mainly the result of a sterile inflammatory response to mobilizing stimuli in the BM microenvironment. In the initiation stage of the mobilization process, HMGB1, which binds to mannan-binding lectin (MBL), regulates the mobilization of HSPCs into peripheral blood (PB) [75].

HMGB1 participates in granulocyte colony-stimulating factor (G-CSF)-induced mobilization of HSCs from the BM into the systemic circulation [76]. Additionally, a clonogenic assay for CFU-granulocyte-monocytes indicated that HMGB1 was required to prevent HSC exhaustion and maintain immune/hematopoietic homeostasis. HMGB1 is linked to substance P (SP) and neurokinin-A (NK-A) to protect the most primitive hematopoietic cells and ensure hematopoietic homeostasis. Mechanistically, HMGB1 negatively regulates hematopoietic stimulation, while SP, a hematopoietic stimulator, decreases HMGB1 expression. Furthermore, NK-A can negatively regulate SP-mediated hematopoietic stimulation [77–79]. The dysfunction of HMGB1 may promote the occurrence and development of hematological malignancies by interfering with the hematopoietic function of the BM.

### HMGB1 and the inflammatory bone marrow microenvironment

The BM is a soft viscous tissue that occupies cavities within the bone [80]. The BM microenvironment is a dynamic network composed of growth factors, cytokines, and stromal cells, which provides a supportive environment for the occurrence and development of hematopoietic malignancies [81]. As a cytokine, HMGB1 can bind to RAGE and TLR4 to activate proinflammatory signaling pathways, such as the NF-κB pathway, and sustain the inflammatory BM microenvironment by inducing cytokine release and recruiting leukocytes. Subsequently, the inflammatory BM microenvironment can accelerate neoplastic transformation and support tumor growth, invasion, and metastases. Infiltrating leukocytes and cancer cells have the ability to secrete HMGB1 in response to hypoxia, injury, inflammatory stimuli, or environmental factors. This loop promotes inflammatory responses and the development of an inflammatory BM microenvironment.

Myeloid-derived suppressor cells (MDSCs) are newly identified immature myeloid cells with immunosuppressive activity. During tumor microenvironment (TME), MDSCs suppress the host anti-tumor immune response through inhibition of T cell proliferation, cytokine secretion, and recruitment of regulatory T cells in hematological malignancies. In all hematological malignancies, several strategies to target MDSCs could improve immune therapies via multiple mechanisms, such as hampering MDSCs function, promoting MDSCs maturation, and depleting MDSCs [82–84]. HMGB1 can facilitate MDSCs differentiation in BM and inhibit the activation of antigen-driven CD4^+^ and CD8^+^ T cells. HMGB1 also increases MDSC-mediated IL-10 production, enhances crosstalk between MDSCs and macrophages, and promotes MDSCs to downregulate the expression of the T cell-homing receptor L-selectin [85]. Circulating complement C1q can stimulate leukocyte-associated Ig-like receptor-1 (LAIR-1) and maintain monocyte quiescence [86]. Very high levels of HMGB1 induce proinflammatory M1-like macrophage differentiation, and high levels of HMGB1 synergize with C1q via RAGE and LAIR-1 to induce the differentiation of monocytes to anti-inflammatory M2-like macrophages [87]. HMGB1 could be released into the BM microenvironment by DCs as a potential immunomodulatory factor to bind with RAGE on the T cell surface and mediate the interaction between DCs and T cells, which is involved in the occurrence and development of hematological malignancies [88]. A study also showed that HMGB1 enhances the maturation and accumulation of DCs by promoting CCR5 and CXCR3 production and inducing potent T cell cytotoxicity [89].

Mesenchymal stem cells (MSCs) play a “double-edged sword” role in hematological malignancies. Studies indicate that MSCs appear to influence pathways that can suppress both proliferation and apoptosis [90]. MSCs protect T cell acute lymphoblastic leukemia (T-ALL) cells from drug-induced apoptosis though mitochondria transfer mechanism, which eventually leads to chemotherapy resistance [91]. Tumor-associated MSCs are essential components of the TME and also associated with a protumorigenic effect by enhancing tumor cell stemness. HMGB1 also regulates MSCs to promote the inflammatory BM microenvironment formation. HMGB1 acts as a chemoattractant to MSCs. Substantial evidences have revealed that HMGB1 significantly upregulates epidermal growth factor receptor (EGFR) and activates the Ras/MAPK pathway to regulate the differentiation of MSCs [92]. These results demonstrate that HMGB1 induces MSCs to secrete multiple cytokines, which are predominantly associated with the development of an inflammatory BM microenvironment. Furthermore, HMGB1 in the inflammatory BM microenvironment can promote the senescence of MSCs via the TLR2/4 and NF-κB signaling pathways, and inhibition of HMGB1 by ethyl pyruvate (EP) can improve lupus nephritis and reverse senescence-associated secretory phenotype (SASP) development [93, 94]. These findings suggest that nuclear HMGB1 can redistribute or relocalize to the extracellular environment in senescent cells. Moreover, senescent fibroblasts secrete oxidized HMGB1, which stimulates cytokine secretion through TLR4 signaling, inducing p53-dependent cellular senescence. Therefore, the alarmin HMGB1 has been considered a central mediator of senescent phenotypes [95]. Interestingly, a recent study found that metformin, a widely used drug for type 2 diabetes, can block HMGB1 translocation and inhibit catabolic production and cell senescence in stem cells (SCs) [96]. Cellular senescence is considered a tumor-suppressive mechanism that permanently arrests cells that are at risk for malignant transformation, can secrete SASP into the BM microenvironment, and transform senescent fibroblasts into proinflammatory cells that have the ability to promote tumor progression [97, 98].

## The role of HMGB1 in hematopoietic malignancies

### Myelodysplastic syndromes

Myelodysplastic syndrome (MDS) is a heterogeneous group of clonal disorders that is characterized by abnormal differentiation of HSCs, ineffective hematopoietic function of BM, and the risk of conversion to acute myeloid leukemia (AML). The inflammatory BM microenvironment is involved in the development and progression of MDS by inducing the apoptotic death of BM progenitor cells. Charoonpatrapong et al. found that DCs released HMGB1 as a potent immunomodulatory cytokine into the BM microenvironment. HMGB1 binds to RAGE on the surface of T cells to mediate the interaction between DCs and T cells [88]. In addition, Velegraki et al. revealed that TLR4 was overexpressed in the BM mononuclear cells of MDS patients compared with those of the control group. TLR4 inhibitors can also inhibit the production of proinflammatory cytokines released by monocytes in patients. Moreover, a study has illustrated that TLR4-dependent inflammatory cytokines not only increase cell apoptosis but also impair the cell clearance capacity of macrophages under the influence of the endogenous ligand HMGB1 [52]. Recently, Angel Y.F. and Kam et al. identified HMGB1 as a previously undescribed target that modulated the innate immune system in MDS. This group used combined siRNAs and the small molecule inhibitor sivelestat to study the loss of function of HMGB1 compared to standard chemotherapy. In MDS cells, sivelestat, a neutrophil elastase inhibitor, increases the expression of PUMA and DNA double-strand breaks and activates caspase-3, which indicates that sivelestat can downregulate HMGB1 and suppress the TLR and NF-κB pathways to promote apoptosis in the BM [99]. The reduction in HMGB1 levels is sufficient to impair MDS cell self-renewal and promote apoptotic cell death. Inhibitors of HMGB1 signaling can provide a first-in-class therapeutic option for patients with MDS and can be used as monotherapy or in combination with chemotherapies to improve the sensitization of MDS cells.

### Acute myeloid leukemia

In acute promyelocytic leukemia (APL), the direct molecular target of all-trans-retinoic acid (ATRA) in human myeloid cells is the PML-RARα oncoprotein that mediates differentiation [100]. It has been proved that upregulated endogenous HMGB1 promoted autophagy and induced NB4 cell differentiation via ubiquitin-binding adaptor protein p62/SQSTM1-mediated degradation of PML-RARα oncoprotein [101]. However, long-term exposure of ATRA and arsenic trioxide (ATO) results in hyper-inflammation and development of the differentiation syndrome (DS) [102]. HMGB1 promoted ATRA/ATO-induced DS by enhancing inflammation through the MEK/ERK signaling pathway [103]. Under the induction of specific chemical reagents, murine erythroleukemia (MEL) cells release HMGBl and promote self-differentiation. However, HMGB1 also mediates the differentiation of MEL cells through pathways other than HMGB1-RAGE [104–107]. During the progression of AML, HMGB1 is secreted to induce TNF-α production and subsequent secretion of IL-1β, which stimulates endothelial cells to release stem cell factor (SCF), which can further promote the proliferation of AML cells. HMGB1 is also dependent on the immune receptor Tim-3 to induce angiogenic VEGF secretion and participate in tumor angiogenesis [69]. Additionally, Liu et al. confirmed that miR-34a suppressed the expression of HMGB1 by directly binding with its 3′-untranslated region (UTR). Overexpression of miR-34a can dramatically reverse apoptosis inhibition by downregulating the expression of HMGB1 in AML [108].

HMGB1 not only is highly expressed and directly induces autophagy in AML cells but also indirectly promotes autophagy to result in therapeutic resistance by enhancing the effect of Beclin-1/PI3KC3 and Atg5-Atg12-Atg16. Reducing the expression of HMGB1 by miR-34a, miR-181b and miR-142-3p enhances the drug sensitivity of AML cells by inhibiting autophagy; moreover, miR-142-3p directly targets HMGB1 to not only represses autophagy but also reduces P-gp to enhance the drug sensitivity of AML cells [108–110]. HMGB1 also increases the expression of monocyte chemoattractant protein 1 (MCP1) and myeloid cell leukemia 1 (Mcl1) to promote the migration of human leukemia monocytic THP1 cells, which is inhibited by glycyrrhizin (GL) [111]. It has been reported that HMGB1 knockdown reduces the expression of RAGE, which is developed from a cell adhesion molecule family and acts as an adhesion molecule in mammalian cells [112]. Extracellular HMGB1 is not only an important DAMP that is released by cells upon necrosis but also a regulatory factor to prevent AML cell necroptosis. When Z-VAD-fmk inhibits caspase activity, etoposide induces necroptosis by triggering cIAP1/2 depletion [113]. However, extracellular HMGB1 prevents this necroptosis. Interestingly, HMGB1 enhances cell viability and regulates necroptosis through the NF-κB pathway rather than preventing cIAP1/2 degradation [114].

### Chronic myeloid leukemia

In chronic myeloid leukemia (CML), HMGB1 knockdown can arrest the cell cycle at the G1 phase and inhibit cell proliferation by downregulating the expression of cyclooxygenase-2 (COX-2) [115]. COX-2 activates the Akt/survivin- and Akt/ID3 signaling pathways, which are related to promoting proliferation [116]. Second-generation tyrosine kinase inhibitors (TKIs), such as dasatinib, can reactivate the induction of apoptotic cell death in patients with imatinib-refractory CML and Philadelphia chromosome-positive ALL (Ph^+^ALL). However, HMGB1-mediated necroptosis gives rise to dasatinib-induced cardiotoxicity, which reduces the clinical applications of dasatinib, indicating that targeting HMGB1 may be a viable strategy to prevent dasatinib-induced cardiotoxicity [117]. A tetrahydrobenzimidazole derivative TMQ0153 has a strong pro-oxidant effect against imatinib-sensitive and imatinib-resistant CML cells. TMQ0153 treatment significantly stimulates the release of HMGB1, leading to immunogenic cell death (ICD), which is a form of chemotherapy-induced tumor cell death [118]. Yang et al. found that cytoplasmic HMGB1 reduced the sensitivity of CML cells to death induced by anticancer drugs by upregulating the autophagy pathway. HMGB1 overexpression increases the transcriptional activity of JNK, ERK, and Beclin-1 [119]. Chen et al. found that HMGB1 knockdown promoted the apoptosis of K562 cells by increasing Bax protein and reducing Bcl-2 protein [115]. Conversely, HMGB1 overexpression inhibits ADM-induced apoptosis in K562 cells by regulating Bcl-2 protein levels and the activity of caspase-3/9 [120]. Moreover, knockdown of HMGB1 significantly inhibits the adhesion of K562 cells [115].

### Acute lymphocytic leukemia

Vincristine, corticosteroids, and l-asparaginase in conjunction with intrathecal therapy can completely alleviate 95% of ALL in patients [121]. Compared with the healthy control and ALL patients with complete remission, serum HMGB1 level of ALL patients was increased, but there was no significant difference in HMGB1 level between the healthy control group and ALL complete remission group, indicating that serum HMGB1 is a useful biomarker to evaluate the prognosis of childhood ALL. Moreover, HMGB1 may be associated with the stages of hemocyte differentiation and maturation. HMGB1 is released from ALL cells and promotes inflammation by stimulating leukemic cells to secrete TNF-α through a MAPK-dependent mechanism [122]. This finding indicates that HMGB1 expression is positively correlated with the clinical status of ALL patients. Moreover, the Ulk1-Atg13-FIP200 complex, which is upstream of HMGB1-Beclin1 and PI3KC3-Beclin1 complexes, promotes HMGB1 trafficking and consequently upregulates autophagy. Therefore, targeting the transformation of autophagic complexes or HMGB1 translocation may inhibit autophagy, and thus reverse ALL drug resistance [123]. Unlike common miRNA effects with negative correlations, inhibition of miR-181a expression induces a decrease in HMGB1 protein in T- and B-ALL cells. This suggests that dysregulation of HMGB1, perhaps due to miR-181a dysregulation, promotes leukemogenesis [124]. Anthracycline can induce a tumor-specific immune response through HMGB1 release in the late stage, and play a role in enhancing the antigen expression of dead tumor cells to DCs through the TLR4 receptor in ALL cells [125].

### Lymphoma

The expression level of HMGB1 in many primary lymphomas is higher than the average level in normal lymph nodes, and HMGB1 is only detected in lymphoma cells. There is a correlation between HMGB1 expression and classification [126]. Chronic lymphocytic leukemia (CLL) is the most common subtype of non-Hodgkin lymphoma (NHL) and is mainly characterized by mature small lymphocytes invading PB and lymphoid tissues such as BM, lymph nodes, and the spleen. Jia et al. found that the plasma HMGB1 levels in CLL patients were significantly higher than those of the healthy control group, and the HMGB1 concentration was related to the absolute lymphocyte count. Furthermore, CLL cells passively release HMGB1 through the HMGB1-RAGE/TLR9 pathway and differentiate CD14^+^ monocytes from CLL cells into nurse-like cells (NLCs), thus regulating the microenvironment. The high number of NLCs is related to the short survival time of CLL patients [127]. Cutaneous T cell lymphoma (CTCL), the second most common extranodal NHL, is characterized by clonal accumulation of postthymic T cells residing in the skin and represents a group of diseases such as mycosis fungoides (MF) and Sézary syndrome (SS) [128, 129]. Senda et al. demonstrated that HMGB1 expression in sera is increased in CTCL patients and correlates with serum levels of soluble IL-2 receptor, lactate dehydrogenase, thymus and activation-regulated chemokines, and the number of eosinophils in PB. It was also found that the level of *HMGB1* mRNA in CTCL-injured skin was significantly increased and positively correlated with *IL*-*4*, *IL*-*10*, *IL*-*19*, and *angiogenin* mRNA levels [130]. It has been reported that IL-4, IL-10, and IL-19 are associated with Th2 polarization [131–133]. These results suggest that enhanced HMGB1 expression may contribute to the progression of CTCL through Th2 polarization and promotion of angiogenesis [130]. Notably, Fredholm S. et al. proved that 72% of CTCL patients had pY-STAT3-positive malignant T cells, and staining for eosinophils and the trafficking factor HMGB1 was also positive, which supports HMGB1 as a possible therapeutic target [134].

To evaluate the significance of HMGB1 in patients with T cell lymphoma, a study found that the expression of HMGB1 in 120 cases of T cell lymphoma was significantly higher than that in 40 cases of reactive lymphoid hyperplasia. Furthermore, the positivity rate of HMGB1 was used as an indicator for diagnosing T cell lymphoma in patients with lymph node biopsy. The specificity of this finding was 63.7%, which was significantly associated with malignancy and clinical stage but not gender, age, or tumor location. Elevated expression of HMGB1 may be a potential diagnostic marker for the development and progression of T cell lymphoma [135]. Zhao T et al. demonstrated that rituximab-induced inhibition of STAT3 activity led to an increase in HMGB1 release and a decrease in IL-10 secretion, triggering immune responses and greatly improving the clinical outcome of patients with diffuse large B cell lymphoma (DLBCL), suggesting that indirectly affecting the immune system rather than directly killing cells led to the elimination of DLBCL [136]. Conversely, HMGB1 stimulates DLBCL cell proliferation by activating the Src/ERK pathway, which is inhibited by EP, causing an accumulation of p27 and cell cycle arrest in the G1 to S phase transition. It has been suggested that EP-mediated blockade of the HMGB1-mediated signaling pathway can effectively inhibit the occurrence of DLBCL and disease progression [137]. Moreover, in their studies, HMGB1 plays a dual role in DLBCL as an inflammatory factor that promotes tumorigenesis and as a cytokine that induces immune responses, which further indicates that HMGB1 has a potential application in the pathogenesis and treatment of DLBCL [138].

In anaplastic large-cell lymphomas (ALCLs), Dejean et al. found that HMGB1 could activate the MMP-9, PAR-2, and NF-κB pathways to induce the release of IL-8, which bound to CXCR1 and CXCR2 on the surface of ALK-positive lymphoid cells to promote the proliferation and metastasis of lymphoid cells. After treatment with the HMGB1 inhibitor glycyrrhiza, the invasion and metastatic abilities of lymphoma cells were significantly decreased [139]. Adult T cell leukemia (ATL) patients have high plasma HMGB1 levels compared with normal controls [140]. It has been reported that high plasma HMGB1 levels in patients with ATL are caused by infection with human T cell lymphotropic virus type I (HTLV-I) [141]. In addition, *HMGB1* mRNA is abundantly expressed in HTLV-I-infected T cell lines. The HTLV-I oncoprotein Tax enhances the expression of the *HMGB1* gene at the transcriptional level by interacting with C/EBP and inducing extracellular release of HMGB1 by T cells. These results suggest that HMGB1 is a potential biomarker and a therapeutic target for ATL [140, 142].

### Multiple myeloma

In MM, high expression of HMGB1 is negatively associated with the 3-year survival of MM patients, which may be involved in promoting MM drug resistance. HMGB1 could participate in DNA damage repair and autophagy. In contrast, when HMGB1 is downregulated, the sensitivity of MM cells to dexamethasone (Dex) is enhanced by activating the mTOR pathway to inhibit autophagy and induce apoptosis [143]. Similarly, Gao et al. found that the expression of the lncRNA MALAT-1 and HMGB1 was dramatically increased in patients with untreated MM, while MALAT-1 expression and HMGB1 protein levels in patients with complete remission were significantly decreased. Furthermore, MALAT-1 increases the expression of HMGB1 at the posttranslational level by inducing HMGB1 ubiquitination in MM cells, thereby promoting autophagy and inhibiting apoptosis [29]. In addition, Roy M. et al. revealed that the expression of HMGB1 increased in MM bortezomib-resistant cells, and bortezomib combined with lycorine efficiently resensitized resistant cells to bortezomib. Mechanistically, the proteasomal degradation of the HMGB1 by lycorine inactivates the MEK-ERK pathway, inhibiting Bcl-2 dissociation from Beclin-1 and consequently suppressing autophagy [30]. Therefore, HMGB1 is an important target for MM patients to increase chemotherapy drug sensitivity.

Interestingly, HMGB1 can also participate in other pathological processes in MM. Similar to DAMPs emitted by apoptotic MM cells, HMGB1 fosters an immunogenic microenvironment to promote antitumor immunity. Recently, studies showed that chemotherapeutic agents, such as melphalan and docosahexaenoic acid (DHA), promoted the release of HMGB1 by ICD, leading to an immune response [144, 145]. Moreover, HMGB1 can act as a thrombosis-related biomarker in patients with MM. After Mel-P, bortezomib, and lenalidomide therapies, the plasma concentrations of HMGB1 were reduced in association with the risk of thrombosis [146, 147]. CXCR4 plays an important role in proliferation, invasion, dissemination, and drug resistance in MM [148]. This indicates that its ligand HMGB1 could regulate MM physiological processes. Because of its pivotal role in the progression of MM, HMGB1 is considered one of the most important potential targets for inhibiting tumor growth, metastasis, and drug resistance and optimizing current anti-MM treatment strategies (Table 1).

**Table 1:** Cellular functions of HMGB1 and related interactors in various types of hematopoietic malignancies

Tumor	Sources of HMGB1	Cellular function	Interactors and pathways	Inhibitors	References
MDS	DCs	Interacts with T cells to mediate DCs	RAGE	NR	[88]
	Plasma and BM	Impairs the ability of macrophages to phagocytose apoptotic cells	TLR4	TLR4 inhibitors	[52]
	BM	Modulates the innate immune system and inhibits apoptosis	TLRs and NF-κB pathways	HMGB1 siRNAs and sivelestat	[99]
AML	APL cell line NB4	Mediates autophagy and affects the degradation of PML-RARα	ROS, p62/SQSTM and PML-RARα	NAC	[101]
	APL	Enhances inflammation and promotes ATRA/ATO-induced DS	MEK/ERK pathways	NR	[103]
	MEL cells	Promotes MEL cells differentiation	NR	NR	[106]
	NR	Stimulates AML cells proliferation and angiogenesis	TNF-α and Tim-3	NR	[69]
	AML cells	Represses apoptosis and promotes autophagy and therapeutic resistance	Beclin-1/PI3KC3, Atg5-Atg12-Atg16	MiR-34a, MiR-181b3, MiR-142-3p	[108-110]
	THP cells	Promotes migration	MCP-1 and Mcl-1	GL	[111]
	Mammalian cells	Reduces adhesion	RAGE	NR	[112]
	Extracellular	Prevents necroptosis	NF-κB pathway	NR	[114]
MPN	CML cells	Promotes proliferation	COX-2, Akt/surviving and Akt/ID3 pathways	Cordycepin	[115]
	Cytoplasmic	Decreases CML cells sensitivity to anticancer drugs	JNK, ERK and Beclin-1	NR	[119]
	CML cells	Inhibits apoptosis	Bax, Bcl-2 and ROS	HMGB1 knockdown	[115]
ALL	ALL cells	Promotes inflammation	TNF-α and MAPK	NR	[122]
		Upregulates autophagy and chemoresistance	Ulk1-Atg13-FIP200 complex and Beclin1	NR	[123]
	T- and B-ALL cells	NR	NR	MiR-181a	[124]
	Pyroptosis cells	Induces cytokine release and CRS	IL-6 and GSDME	NR	[125]
CLL	CLL cells	Differentiates monocytes into NLCs	RAGE/TLR9	NR	[127]
CTCL	Peripheral blood	Promotes Th2 polarization and angiogenesis	IL-4, IL-10, IL-19 and angiogenin	NR	[130]
	Extracellular	Stimulates DLBCL cell proliferation	Src/ERK pathway	EP	[137]
ALCLs	Extracellular	Promotes the proliferation and metastasis of lymphoid cells.	NR	GL	[139]
MM	Extracellular	Promotes drug resistance, DNA damage repair and autophagy	NR	NR	[143]
	Nucleus and cytosol	Promotes autophagy and inhibits apoptosis	ubiquitination	LncRNA MALAT-1	[29]
	MM bortezomib-resistant cells	Degrades HMGB1 protein and inhibits autophagy	MEK/ERK pathway	Lycorine	[30]
	Apoptotic MM cells	Fosters an immunogenic microenvironment and promotes antitumor immunity	NR	NR	[144, 145]
	Extracellular	Acts as a thrombosis-related biomarker	NR	NR	[146, 147]

*NR* not reported, *MPN* myeloproliferative neoplasms

## The potential clinical applications of HMGB1

### Hematopoietic stem cell transplantation

Hematopoietic stem cell transplantation (HSCT) is an intensive therapy to treat hematologic malignancies, but graft-versus-host disease (GVHD) is a frequent severe inflammatory complication that is associated with poor outcomes [149]. Yujiri et al. found increased serum levels of HMGB1 in patients who developed acute GVHD (aGVHD) after HSCT, which indicates that HMGB1 may be a useful indicator of GVHD [150]. Additionally, enhanced HMGB1 is reported to promote STAT3 expression in CD4^+^ T cells via modulation of its DNA methylation, subsequently inhibiting Tregs and promoting the Th17 response during GVHD [151]. It has been demonstrated that genetic variations in cytokine genes can modulate immune reactions after HSCT. An inherited variation in HMGB1 is associated with outcomes after allogeneic HSCT (allo-HSCT) [152]. Thus, HMGB1 is likely to play an important role in the development of GVHD, known as the graft-versus-tumor (GVT) effect, and possibly engraftment because of its central role in the activation of APCs and tissue regeneration. Moreover, the compound cyclopentylamino carboxymethylthiazolylindole (NecroX)-7 could protect mice against lethal GVHD by reciprocal regulation of regulatory T/Th1 cells, attenuating systemic HMGB1 accumulation and inhibiting the HMGB1-mediated inflammatory response [153]. Cyclophosphamide (CY) in combination with either ablative doses of total body irradiation (TBI) or the oral alkylating agent busulfan (Bu) is the most common conditioning regimen for allo-HSCT. However, TBI and CY can mobilize HMGB1 to the PB, and increased levels of HMGB1 correlate with increased PAI-1 after allo-HSCT, inducing transplantation-associated coagulopathy (TAC) conditions such as veno-occlusive disease (VOD) [154]. Recombinant human soluble thrombomodulin (rhTM) is used to treat disseminated intravascular coagulation (DIC) caused by aGVHD and significantly decreases HMGB1 [155]. Extracorporeal photopheresis (ECP) depends on infusion of UVA-irradiated and 8 methoxy-psoralen (PUVA)-treated leukocytes and is an effective treatment measure for GVHD. In vitro PUVA treatment induces the expression of HMGB1 in dying T cells, especially upon T cell activation, leading to their phagocytosis by macrophages and DCs [156]. In AML patients who received allo-HSCT, a higher γδ T cell count, which is an important early source of TNF-α and IFN-γ, predicted a better prognosis. A recent study found that PD-1^+^TIM-3^+^ Vδ2 T cells, PD-L1, and HMGB1 were significantly higher in AML patients than in healthy controls, suggesting that PD-1 alone is insufficient to indicate functional impairment, and Vδ2 T cells may require anti-TIM-3 inhibition for functional revival [157].

### HMGB1 and chemoresistance in hematopoietic malignancies

**Fig. 4:**
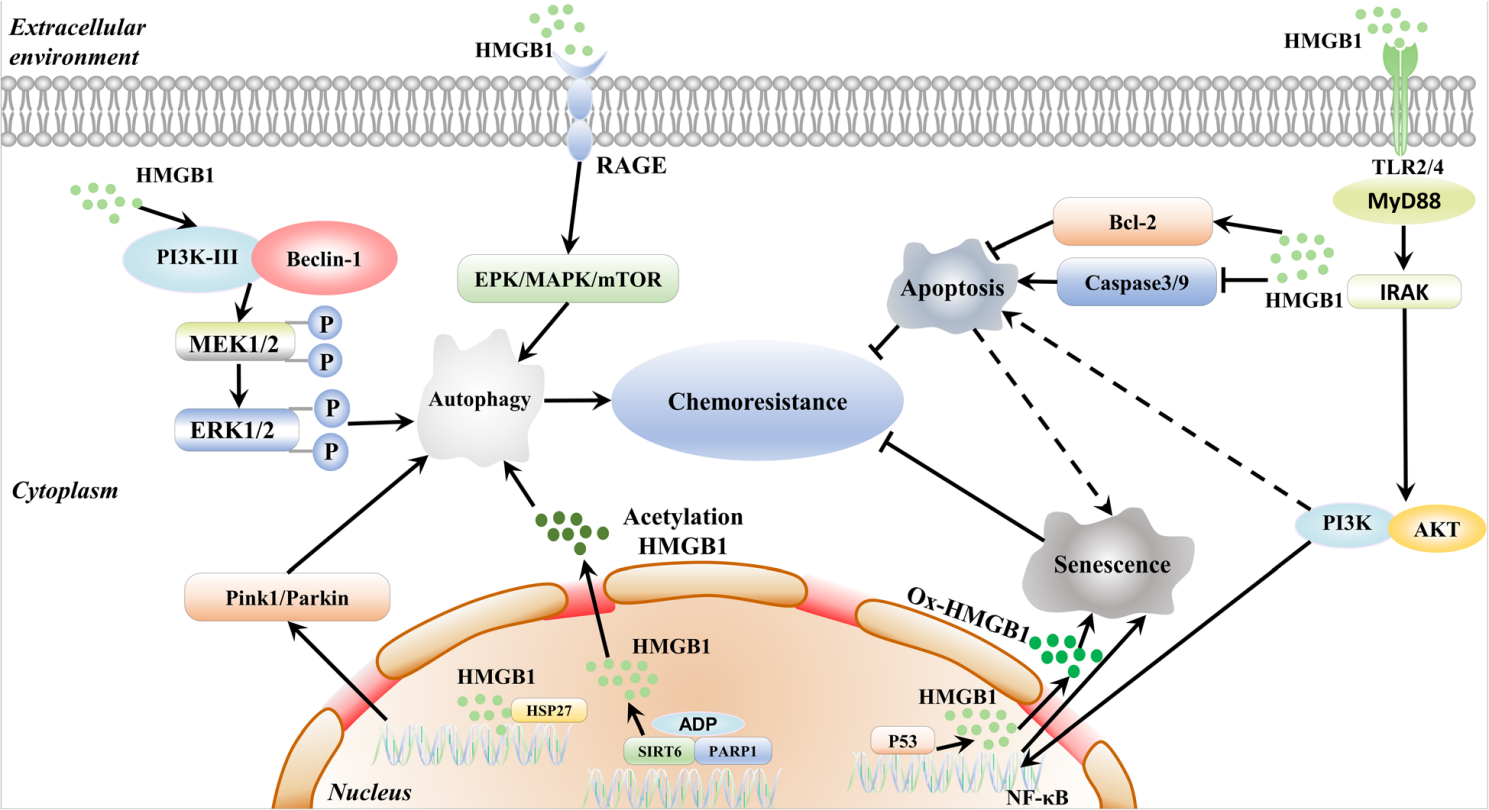
The dual role of HMGB1 in chemoresistance. HMGB1-dependent autophagy promotes chemoresistance in three ways: nuclear HMGB1 upregulates the expression of HSP27, cytoplasmic HMGB1 activates the Beclin-1/PI3K-III complex, and extracellular HMGB1 binds to RAGE. Chemotherapy also activates SIRT6/PARP1 and promotes HMGB1 acetylation and translocation, inducing autophagy. HMGB1 inhibits apoptosis to enhance chemoresistance by inhibiting the caspase3/9 pathway and inducing Bcl-2 release. In contrast, HMGB1 induces tumor cell senescence to improve chemotherapy. HMGB1 binds to TLR2/4 and then activates the NF-κB signaling pathway, inducing p53-dependent cellular senescence. HMGB1 can also induce apoptosis-to-senescence in tumor cells

Acquired chemoresistance is a major obstacle in the clinical treatment of hematological malignancies. Many studies have demonstrated that chemotherapy agents including docetaxel, doxorubicin (DNR), cisplatin, etoposide, and methotrexate induce HMGB1 upregulation and promote cytosolic HMGB1 translocation [158–160]. Moreover, DNR, vincristine (VCR), etoposide (VP-16), cytosine arabinoside (Ara-C), adriamycin (ADM), and ATO can increase HMGB1 expression and promote chemoresistance in hematological malignancies [22, 44]. Downregulating HMGB1 inhibits autophagy and enhances bortezomib activity in MM [30]. HMGB1 is becoming a recognized therapeutic target for chemotherapy resistance (Fig. 4) [161].

#### Autophagy

Autophagy is a degradation mechanism that alters cells to restore their energy balance during periods of varying nutrient availability [162]. However, autophagy improves the survival of cancer cells at the later times under stressful conditions, such as nutrient depletion, hypoxia, and therapeutic damage [163]. Blocking autophagy could increase cancer cells sensitivity to chemotherapy. For instance, bortezomib-induced MCL cell death was significantly potentiated by compounds that interfered with autophagosomal function [164]. HMGB1-dependent autophagy promotes chemotherapy resistance in three ways: nuclear HMGB1 upregulates the expression of HSP27, cytoplasmic HMGB1 activates the Beclin-1/PI3K-III complex, and extracellular HMGB1 binds to RAGE [45, 165]. By targeting HMGB1, autophagy inhibition is a potential therapeutic strategy for hematopoietic malignancies [166, 167]. Nuclear HMGB1 can activate the HSP27 pathway during autophagy, and the Pink1/Parkin pathway is required for HMGB1/HSP27-dependent mitophagy. The HSP27 pathway may be a potential mechanism by which HMGB1 regulates nuclear autophagy [168, 169]. In autophagy-related chemoresistance, the dissociation and recoupling of autophagic complexes are essential events. HMGB1 gene transfection can increase the LC3-II level and inhibit the rapamycin complex 1 (mTORC1) pathway to strongly induce autophagy and promote chemoresistance in leukemia cells [170, 171]. HMGB1 is released from dying cancer cells and enhances autophagy-induced chemoresistance and regrowth via RAGE-mediated ERK/Drp1 phosphorylation. HMGB1 and RAGE inhibitors abolish Drp1 phosphorylation and significantly enhance sensitivity to chemotherapeutic treatment by suppressing autophagy [172]. Moreover, treating leukemic cells with chemotherapeutic drugs leads to the translocation of HMGB1, which is involved in autophagy and ultimately promotes chemoresistance in leukemia. Chemotherapy-induced ADP-ribosylation activates SIRT6 and PARP1 and then promotes HMGB1 acetylation and translocation, finally resulting in chemotherapy-induced autophagy in leukemic cells [22, 24].

#### Apoptosis inhibition

Apoptosis generally occurs through two different pathways: the internal pathway and the external pathway. HMGB1 inhibits both apoptosis pathways, thereby enhancing chemoresistance in cancer cells. HMGB1 can inhibit the caspase3/9 pathways, releasing proapoptotic initiators (Bax-Bak) and inducing the expression of antiapoptotic proteins (Bcl-2) [173]. For example, HMGB1 inhibits apoptosis in leukemia K562 cells by regulating the protein level of Bcl-2 and the activity of caspase-3 and caspase-9 [120]. It was also found that inhibition of HMGB1 with siRNAs and sivelestat could activate caspase-3 and promote MDS cell death [99].

#### Senescence

HMGB1 binds to TLR2/4 and then activates the NF-κB signaling pathway to induce p53-dependent cellular senescence [94, 95]. Interestingly, the HMGB1 protein is a double-edged sword. As an antiapoptotic protein, HMGB1 promotes chemotherapy resistance. However, as an enhancer of senescence, HMGB1 induces tumor cells to undergo an apoptosis-to-senescence shift to improve chemotherapy effectiveness [174]. This suggests that HMGB1 could be a target for selectively enforcing tumor suppression. These findings provide new insights into the mechanism of resistance to chemotherapy drugs.

### The therapeutic strategies to inhibit HMGB1 in cancer

To date, several strategies have been proposed to directly or indirectly inhibit HMGB1 expression, release, and activity to treat hematopoietic malignancies (Table 2).

**Table 2:** The effects of various HMGB1 inhibitors

Compound	Type of studies	Biological function	References
HMGB1-neutralizing antibody	In vitro	Inhibits HMGB1-induced autophagy and increases the sensitivity of leukemia cells to chemotherapy	[175]
mAb (2G7)	In vivo	Improves arthritis, LN and drug-induced liver injury	[176-178]
s-RAGE	In vivo	Blocks the HMGB1-RAGE signaling pathway	[179]
HMGB1 A-box	In vitro	Inhibits the proinflammatory actions of the B-box	[5]
TAT-HMGB1A	In vitro	Reduces secretion of endogenous HMGB1 protein	[180]
GL	In vitro, in vivo	Suppresses HMGB1 phosphorylation and secretion via PKC/CaMKIV	[181]
EP	In vitro, in vivo	Inhibits HMGB1 secretion by inducing HO-1 via PI3k/Akt and Nrf2 pathways; reverses the HMGB1-induced senescent phenotype of BM-MSCs; reduces RAGE expression and NF-κB/STAT3 pathway activation	[93, 184, 185]
quercetin	In vitro	Promotes apoptosis by attenuating the expression of HMGB1 and RAGE and suppressing the activation of NF-κB	[186]
ICM	In vitro	Inhibits HMGB1 nucleoplasmic translocation and autophagy by enhancing the interaction between Beclin-1 and E3 ubiquitin ligase RNF216	[187]
sLPC	In vivo	Suppresses HMGB1 phosphorylation and extracellular release	[188]
P5779	In vitro, in vivo	Interrupts disulfide-HMGB1/MD-2 binding; suppresses HMGB1-induced TNF release	[189]
rTM	In vitro, in vivo	Decreases serum HMGB1 levels and improves SIRS in hematological malignancies; improves DIC in AML; inhibits HMGB1 protein secretion and inhibits I-κB phosphorylation	[190-192]

#### Anti-HMGB1 antibodies

Administration of a polyclonal HMGB1-neutralizing antibody inhibited HMGB1-induced autophagy and increased the sensitivity of leukemia cells to chemotherapy, suggesting that HMGB1 is a potential drug target for therapeutic interventions [175]. Several anti-HMGB1 monoclonal antibodies have been developed for clinical applications. The monoclonal antibody 2G7 binds to the HMGB1 epitope containing aa 53-63 and has shown beneficial therapeutic effects in experimental models of arthritis, lupus nephritis (LN) and drug-induced liver injury [176–178].

#### Targeting receptors

Soluble RAGE (s-RAGE) is an endogenous cleaved soluble form of RAGE that blocks the HMGB1-RAGE signaling pathway in animal tumor models and has decoy receptor properties [179]. Recombinant HMGB1 A-box efficiently interacts with RAGE, competing with the HMGB1 protein to bind to RAGE, and this peptide lacks the proinflammatory cytokine activity of the B-box [5]. TAT-labeled HMGB1 A-box-His6 (TAT-HMGB1A) was used as a pharmaceutical protein ex vivo and significantly reduced the secretion of endogenous HMGB1 protein by structurally modulating its cellular membrane penetration [180].

#### Small molecules

GL, a triterpenoid saponin glycoside of glycyrrhizic acid, specifically binds to both HMG boxes of the HMGB1 cytokine, inhibiting HMGB1-induced proliferation and migration, as well as the formation of blood vessels, and reducing HMGB1-stimulated inflammatory conditions [181]. GL also suppresses HMGB1 phosphorylation and secretion by reducing the interaction between HMGB1 and protein kinase C (PKC) or calcium/calmodulin-dependent protein kinase IV (CaMKIV) [182]. EP, an anti-inflammatory factor, directly chelates calcium and inhibits HMGB1 phosphorylation and secretion [183]. EP also attenuates the active secretion of HMGB1 by inducing heme oxygenase-1 (HO-1) expression via activation of the PI3K/Akt and Nrf2 pathways [184]. Moreover, EP reverses the HMGB1-induced senescent phenotype of BM-MSCs and prolongs the survival of MRL/lpr mice [93]. EP also impairs HMGB1 secretion, leading to reduced RAGE expression and NF-κB/STAT3 pathway activation [185]. As an antioxidant, quercetin promotes apoptosis by attenuating the expression of HMGB1 and RAGE and suppressing the activation of NF-κB in MCF-7 cells [186]. A novel type of HMGB1 secretion inhibitor, erythropoietin (inflachromene, ICM), increases the ubiquitination of Beclin-1 by enhancing the interaction between Beclin-1 and the E3 ubiquitin ligase RNF216, inhibiting HMGB1 nucleoplasmic translocation and thereby inhibiting autophagy [187]. Stearoyl lysophosphatidylcholine (sLPC), a traditional Chinese medicine ingredient, suppresses HMGB1 phosphorylation and inhibits LPS-induced extracellular release of HMGB1 through the G2A/calcium/CaMKKβ/AMPK pathway [188].

#### Small peptides and peptidomimetics

A peptide inhibitor of HMGB1 (P5779) that selectively interrupts disulfide-HMGB1/MD-2 binding without inhibiting other TLR4/MD-2 ligands has been identified [35]. Recently, it was demonstrated that folic acid mimics the binding of P5779 at the intersection of TLR4 and MD-2. These folic acid-derived P5779 mimetics inhibit HMGB1-induced TNF release in human macrophages [189]. Recombinant human thrombomodulin (rTM) significantly decreases serum HMGB1 levels and improves systemic inflammatory response syndrome (SIRS) in patients with hematological malignancies [190]. In AML patients, rTM can successfully treat DIC, which is correlated with platelet-derived HMGB1 [191, 192]. Moreover, rTM administration inhibits HMGB1 protein secretion and the activation of NF-κB by inhibiting I-κB phosphorylation [193, 194].

## Conclusions and perspectives

HMGB1 has been confirmed to exert various effects on pathological symptoms and different stages of hematological malignancies. HMGB1 may be a very useful biomarker for the diagnosis and prognosis of hematological malignancies. Moreover, HMGB1 is related to the chemoresistance of various hematological malignancies. To date, the PTM of HMGB1 in the context of various hematological malignancies remains mostly unexplored; likewise, the redox forms of HMGB1 that are involved in hematological malignancies have not been revealed in detail. This has opened up promising new avenues of investigation in these fields.

Although HMGB1 has an important impact on hematopoietic regulation of HSCs and the inflammatory BM microenvironment, it is not clear how HMGB1 is involved in the development of HSCs into hematological malignancies. Furthermore, HMGB1 is released into the extracellular environment from activated immune cells or passively released from damaged or necrotic cells. Extracellular HMGB1 is a risk factor for a series of hematological malignancies. Likewise, HMGB1 is not only an antiapoptotic protein but also an enhancer of senescence, and plays a dual role in the regulation of drug resistance in leukemia cells. The application of targeting antibodies or biological inhibitors of HMGB1 as therapeutic drugs is still confronted with a variety of challenges.

In conclusion, it is necessary to further understand the mechanism by which extracellular and nuclear HMGB1 affects HSCs and the BM microenvironment and how to maximize its therapeutic potential in different hematological malignancies.

## Data Availability

Not applicable, all information in this review can be found in the reference list.

## Abbreviations

ADM: Adriamycin
aGVHD: Acute GVHD
ALCLs: Anaplastic large-cell lymphomas
allo-HSCT: Allogeneic HSCT
AML: Acute myeloid leukemia
Ara-C: Cytosine arabinoside
ATO: Arsenic trioxide
ATRA: All-trans-retinoic acid
AP1: Activator protein 1
APL: Acute promyelocytic leukemia
ATL: Adult T cell leukemia
BM: Bone marrow
Bu: Busulfan
CaMKIV: Calcium/calmodulin-dependent protein kinase IV
CBP: CREB-binding protein
CLL: Chronic lymphocytic leukemia
CML: Chronic myeloid leukemia
COX-2: Cyclooxygenase-2
CRM-1: Chromosome-region maintenance-1
CTCL: Cutaneous T cell lymphoma
CXCL: CXC motif ligand 12
CXCR4: CXC motif chemokine receptor type 4
CY: Cyclophosphamide
DAMPs: Damage-associated molecular pattern molecules
DCs: Dendritic cells
Dex: Dexamethasone
DIC: Disseminated intravascular coagulation
DLBCL: Diffuse large B cell lymphoma
DNR: Doxorubicin
ds-HMGB1: Disulfide HMGB1
ECP: Extracorporeal photopheresis
EGFR: Epidermal growth factor receptor
EP: Ethyl pyruvate
ERK1/2: Extracellular signal-regulated kinase 1 and 2 (ERK1/2)
fr-HMGB1: Fully reduced HMGB1
G-CSF: Granulocyte colony-stimulating factor
GL: Glycyrrhizin
GPCR: G-protein-coupled seven-transmembrane receptor
GVHD: Graft-versus-host disease
GVT: Graft-versus-tumor
HMG: High mobility group protein
HMGB1: High-mobility group box 1
HO-1: Heme oxygenase-1
HSCs: Hematopoietic stem cells
HSCT: Hematopoietic stem cell transplantation
HTLV-I: Human T cell lymphotropic virus type I
ICD: Immunogenic cell death
ICM: Inflachromene
IFN: Interferon
IgV: Immunoglobulin variable
IL-6: Interleukin-6
LAIR-1: Leukocyte-associated Ig-like receptor-1
LN: Lupus nephritis
LRRs: Leucine-rich repeats
MAPK: Mitogen-activated protein kinase
MBL: Mannan-binding lectin
Mcl1: Myeloid cell leukemia 1
MCP1: Monocyte chemoattractant protein 1
MDS: Myelodysplastic syndrome
MDSC: Myeloid-derived suppressor cell
MEFs: Mouse embryonic fibroblasts
MEL: Murine erythroleukemia
MF: Mycosis fungoides
MM: Multiple myeloma
M-MDSC: Monocytic-MDSC
MSCs: Mesenchymal stromal cells
mTOR: Rapamycin
mTORC1: Rapamycin complex 1
NecroX: Cyclopentylamino carboxymethylthiazolylindole
NESs: Nuclear export signals
NHL: Non-Hodgkin lymphoma
NK cells: Natural killer cells
NK-A: Neurokinin-A
NLCs: Nurse-like cells
NLSs: Nuclear localization sequences
OA: Okadaic acid
ox-HMGB1: Oxidized HMGB1
p300: Histone acetyltransferase p300
PB: Peripheral blood
PBMCs: Peripheral blood mononuclear cells
PCAF: P300/CBP-associated factor
PD1: Programmed cell death 1
Ph^+^ALL: Philadelphia chromosome-positive acute lymphoblastic leukemia
PKC: Protein kinase C
PPAR-γ: Peroxisome proliferator-activated receptor gamma
PRRs: Pattern recognition receptors
PtdSer: Phosphatidylserine
PTMs: Posttranslational modifications
PUVA: 8 Methoxy-psoralen
RAGE: Advanced glycation end products
rhTM: Recombinant human soluble thrombomodulin
rTM: Recombinant human thrombomodulin
SASP: Senescence-associated secretory phenotype
SCF: Stem cell factor
SCs: Stem cells
SDF-1: Stromal cell-derived factor-1
SIRS: Systemic inflammatory response syndrome
sLPC: Stearoyl lysophosphatidylcholine
SP: Substance P
s-RAGE: Soluble RAGE
SS: Sézary syndrome
STAT1: Signal transducer and activator of transcription 1
TAC: Transplantation-associated coagulopathy
TAT-HMGB1A: TAT-labeled HMGB1 A-box-His6
T-ALL: T cell ALL
TBI: Total body irradiation
TIM-3: T cell immunoglobulin mucin-3
TIR: Toll/interleukin-1 receptor
TKIs: Tyrosine kinase inhibitors
TLR4: Toll-like receptor 4
TME: Tumor microenvironment
TNF-α: Tumor necrosis factor alpha
Treg cells: Regulatory T cells
Ub: Ubiquitin
UPS: Ubiquitin proteasome system
UTR: Untranslated region
VCR: Vincristine
VEGF: Vascular endothelial growth factor
VOD: Veno-occlusive disease
VP-16: Etoposide
